# Quality Assessment of Virus-Like Particles at Single Particle Level: A Comparative Study

**DOI:** 10.3390/v12020223

**Published:** 2020-02-17

**Authors:** Irene González-Domínguez, Eduard Puente-Massaguer, Laura Cervera, Francesc Gòdia

**Affiliations:** Departament d’Enginyeria Química Biològica i Ambiental, Universitat Autònoma de Barcelona, Cerdanyola del Vallès, 08193 Barcelona, Spain; laura.cervera@uab.cat (L.C.); francesc.godia@uab.cat (F.G.)

**Keywords:** VLP, viral quantification, NTA, flow virometry, SRFM, cryo-TEM, SEM

## Abstract

Virus-like particles (VLPs) have emerged as a powerful scaffold for antigen presentation and delivery strategies. Compared to single protein-based therapeutics, quality assessment requires a higher degree of refinement due to the structure of VLPs and their similar properties to extracellular vesicles (EVs). Advances in the field of nanotechnology with single particle and high-resolution analysis techniques provide appealing approaches to VLP characterization. In this study, six different biophysical methods have been assessed for the characterization of HIV-1-based VLPs produced in mammalian and insect cell platforms. Sample preparation and equipment set-up were optimized for the six strategies evaluated. Electron Microscopy (EM) disclosed the presence of several types of EVs within VLP preparations and cryogenic transmission electron microscopy (cryo-TEM) resulted in the best technique to resolve the VLP ultrastructure. The use of super-resolution fluorescence microscopy (SRFM), nanoparticle tracking analysis (NTA) and flow virometry enabled the high throughput quantification of VLPs. Interestingly, differences in the determination of nanoparticle concentration were observed between techniques. Moreover, NTA and flow virometry allowed the quantification of both EVs and VLPs within the same experiment while analyzing particle size distribution (PSD), simultaneously. These results provide new insights into the use of different analytical tools to monitor the production of nanoparticle-based biologicals and their associated contaminants.

## 1. Introduction

Virus-like particles (VLPs) are considered a promising platform in the field of vaccine development. Nowadays, there are several licensed VLP-based vaccines, such as Cervarix^®^, Gardasil^®^, Hecolin^®^ or Porcilis PCV^®^ and more than 100 candidates are undergoing clinical trials [1]. Their success as immunogens lies on their ability to mimic native viruses without containing a viral genome. Their highly organized and repetitive antigen structure has shown effective cellular and humoral immune responses [2]. Furthermore, advances in the field of bioengineering have widened their possible applications; VLP technology accepts several modifications including encapsulation, chemical conjugation or genetic engineering. By doing so, VLPs can be pseudotyped or used either as DNA or drug nanocarriers [1,3].

VLP quality assessment is of major importance since both, the physicochemical and biological properties, are responsible of their clinical efficacy. The preservation of their structural integrity during all the stages of vaccine manufacturing, storage and administration is critical to ensure their success [4]. The study of particle size distribution (PSD) or particle concentration are some of the critical quality attributes (CQA) that could be monitored in this regard [5]. Overall, the specific detection and quantification of VLPs entails several difficulties, especially for enveloped VLPs, which are composed of a protein capsid surrounded by the host-cell lipid membrane. VLPs must be distinguished from other similar nanovesicle structures; extracellular vesicles (EVs), including exosomes and microvesicles [6], adventitious viruses, or baculoviruses (BV) in insect cell systems [7], are important process-related impurities. In this sense, traditional quantification methods such as TCID_50_ or PCR have a limited applicability due to the non-infective nature of VLPs.

Comprehensive studies on VLP-based vaccine candidates have been conducted by multiple approaches, including biochemical, biological and biophysical methods [3]. Biochemical protein gels, biological enzyme-linked immunosorbent assay (ELISA) or immunoblot are normally used [8,9,10,11]. Nonetheless, these assays cannot distinguish assembled from non-assembled structures [12]. Among biophysical methods, analytical ultracentrifugation, dynamic light scattering (DLS) and transmission electron microscopy (TEM) are the reference methods used to assess VLP physical properties [3]. Recently, technical progress in the field of microscopy, as well as the application of nanotechnology to virology, have given rise to several single nanoparticle analytical technologies. These techniques represent the most advanced methods to evaluate VLP size, polydispersity, purity and even nanoparticle composition simultaneously [3,12]. 

Among them, electron microscopy (EM) has traditionally been the preferred technique since resolution at the nanometric or even atomic level is achieved [13]. Within EM methods, transmission (TEM), scanning (SEM) and cryogenic (cryo-TEM) methodologies are frequently used. TEM is the gold standard technique for the characterization of virus-like structures as reported in a myriad of studies [3,14]. This methodology requires a contrast medium for sample visualization, typically a heavy metal solution containing a cationic or anionic salt, being negative staining the most extended strategy [15]. In TEM-Negative staining, a thin layer of biological material is covered by a dried non-crystalline amorphous layer of a heavy metal salt, typically uranyl acetate. Differential electron scattering between the biological material and the surrounding staining layer enables the visualization of the specimen. The application of SEM in the characterization of different materials has been demonstrated in several works [16]. However, few studies address its use as a tool for VLP characterization. The addition of Alcian Blue solution to the grid before sample deposition results in the activation of the grid with a net positive charge, with successful results reported for the visualization of other negatively charged specimens such as nucleic acids [17]. Since viral structures and EVs are known to have an overall negative charge at physiological pH [18,19], this strategy could improve their adsorption and reduce nanoparticle loss during the sample preparation process. Cryo-TEM has also gained increasing interest as a tool for nanoparticle visualization over the last years [13]. Essentially, this technique enables the visualization of viruses and VLPs in their native conformation at nanometric and even atomic scale [3], and the addition of a contrast solution is not required. A key point of this technique is the rapid freezing process which reduces sample damage. Therefore, the selection of an adequate grid and support film is of upmost importance since the correct formation of a thin ice film is pivotal for an adequate sample visualization. Perforated carbon films are generally the preferred option since they allow the biological material to be imaged in the ice generated between holes in the carbon support film [20,21].

The study of viral vectors and nanoparticles by confocal microscopy has been traditionally restricted by the Abbe diffraction limit. However, the appearance of super-resolution fluorescence microscopy (SRFM) enabling to surpass this constraint has opened a breadth of opportunities to apply confocal microscopy to the nanoscale [22]. Despite SRFM has been mainly used to study cellular processes, its application to appraise individual viral structures is becoming more popular [23]. In previous works, a method for VLP quantification by HyVolution2 SRFM has been described by González-Domínguez and co-workers [24,25]. This method combines sub-Airy confocal microscopy with mathematical deconvolution, which has been described to achieve resolutions up to 140 nm [26]. Finally, light scatter-based technologies, such as nanoparticle tracking analysis (NTA) and flow virometry are also gaining attention for viral particle and EV quantification [5,12,27,28]. NTA is a method to characterize and quantify nanoparticles in solution that relates the rate of Brownian motion to nanoparticle size. Its use in the assessment of nanoparticles has been reported for viruses, VLPs and other nanoparticles [5,29,30,31]. This technique is theoretically able to detect nanoparticles with a size comprised between 30 and 1000 nm, but the nanoparticle concentration has to be maintained around 10^8^ particles/mL and 20–60 particles/frame [29]. The latter indicates that the range of possible nanoparticle concentrations is narrow, and it is often required to dilute the sample to meet this criterion, which is generally based on trial-and-error. Flow virometry has recently emerged as a technique to specifically detect viruses similarly to conventional cell-based flow cytometry [27]. Labeling studies at single particle level, particle quantification or virus sorting are some of the applications that can be performed with this technology [27]. Considering the high difference in volume between a cell and a nanoparticle, which can be one million-fold [32], the acquisition settings need to be adjusted to detect the scattered or fluorescence signal from nanoparticles. Still, a significant loss of scattered light that fall in the range of the background noise of the instrument and different sensitivities between equipments are a general concern [33]. To address this issue, the implementation of the violet (405 nm) side scatter (V-SSC) has been reported to improve the sensitivity but also the resolution of the technique [34]. Owing to the specific features of each analytical method, characterization results such as particle concentration obtained by different techniques are often difficult to compare. 

The aim of this work is the characterization of VLPs using several advanced nanoparticle analytical methods, and to discuss the technological limitations that may affect their use, including sample preparation or equipment set-up. The study of PSD, particle ultrastructural analysis, VLP quantification and differentiation from other nanoparticle subpopulations has been performed. The VLPs analyzed in this work are HIV-1 Gag VLPs, which are a promising platform for the development of a vaccine candidate against HIV, but also as a scaffold for chimeric or multivalent vaccine development [2,35]. Upon expression in the host cell, the Gag polyprotein travels to the cell membrane and after an oligomerization process, HIV-1 Gag VLPs are released from the cell through a budding process [36,37]. Thus, the final nanoparticles are enveloped by the host cell lipid membrane [38], with sizes comprised between 100–200 nm. In previous works, the Gag polyprotein has been fused to GFP to track the VLP production process [36]. By doing so, VLPs could be easily quantified and distinguished from other contaminant particles. Besides, product characteristics are known to be affected by the expression system selected for VLP production [39]. Here, the two most relevant systems for the generation of HIV-1 Gag-based VLPs have been used [40]. VLPs obtained by transient gene expression in HEK 293 cells and baculovirus (BV) infection in Sf9 cells have been characterized in parallel. This study provides relevant data on the use of different analytical methods to evaluate the production of VLPs and their associated contaminants in animal cell-based bioprocesses. 

## 2. Materials and Methods

### 2.1. HEK 293 Mammalian Cell Line, Culture Conditions and Transient Transfection

The mammalian cell line used in this work is a serum-free suspension-adapted HEK 293 cell line (HEK 293SF-3F6 from NRC, Montreal, QC, Canada) kindly provided by Dr. Amine Kamen from McGill University (Montreal, QC, Canada). Cells were cultured as previously described [36]. HIV-1 Gag-eGFP VLP production was achieved by transient transfection. Briefly, the pGag-eGFP plasmid encoding for the Gag-eGFP polyprotein, is diluted with FreeStyle 293 medium (Invitrogen, Carlsbad, CA, USA) and vortexed for 10 s, then polyethylenimine (PEI) is added at 1:2 DNA:PEI ratio (*w*/*w*) and vortexed three times, the mixture is incubated for 15 min at RT and is added to the cell culture, where a medium exchange has been already performed. HIV-1 Gag-eGFP VLPs were harvested at 72 h post transfection (hpt) by centrifugation at 1000× *g* during 15 min. Supernatants were stored at 4 °C until analysis. Non-transfected negative controls reproducing cell growth conditions were also produced, for comparison.

### 2.2. Sf9 Insect Cell Line, Culture Conditions and Baculovirus Infection

The suspension-adapted lepidopteran insect cell line used in this work is the *Spodoptera frugiperda* (Sf9, cat. num. 71104, Merck, Darmstadt, Germany) gently provided by Dr. Berrow (Institute of Biomedical Research, Barcelona, Spain). Sf9 cells were cultured in Sf900III medium (Thermo Fisher Scientific, Grand Island, NY, USA) in 125-mL disposable polycarbonate Erlenmeyer flasks [25]. Cell cultures were shaken at 130 rpm using an orbital shaker (Stuart, Stone, United Kingdom) and maintained at 27 °C. HIV-1 Gag-eGFP VLP production was achieved through infection with the recombinant baculovirus *Autographa californica* multiple nucleopolyhedrovirus (*Ac*MNPV) (BD Biosciences, San José, CA, USA) enconding for the Gag-eGFP protein. Shortly, Sf9 cells were grown to 2–3 × 10^6^ cells/mL and were infected at a multiplicity of infection (MOI) of 1. HIV-1 Gag-eGFP VLPs were harvested at 40 or 72 h post infection (hpi) by centrifugation at 1000× *g* during 15 min, and supernatants were kept at 4 °C until analysis. Non-infected negative controls reproducing cell growth conditions were also produced, for comparison.

Biophysical analyses were performed on HIV-1 Gag-eGFP VLP supernatants obtained as previously described. FreeStyle and Sf900III cell culture media and conditioned cell culture media obtained from HEK 293 and Sf9 non-transfected/infected conditions were also assessed in each analysis as negative controls. 

### 2.3. Electron Microscopy (EM)

EM analyses were performed at Servei de Microscòpia at Universitat Autònoma de Barcelona (UAB, Barcelona, Spain). Particle size distribution (PSD) analyses were performed with ImageJ Fiji (ImageJ, NIH, WI, USA) and SigmaPlot 12.0 (Systat Software Inc., San Jose, CA, USA).

#### 2.3.1. Transmission Electron Microscopy (TEM)-Negative Staining

**Figure 1:**
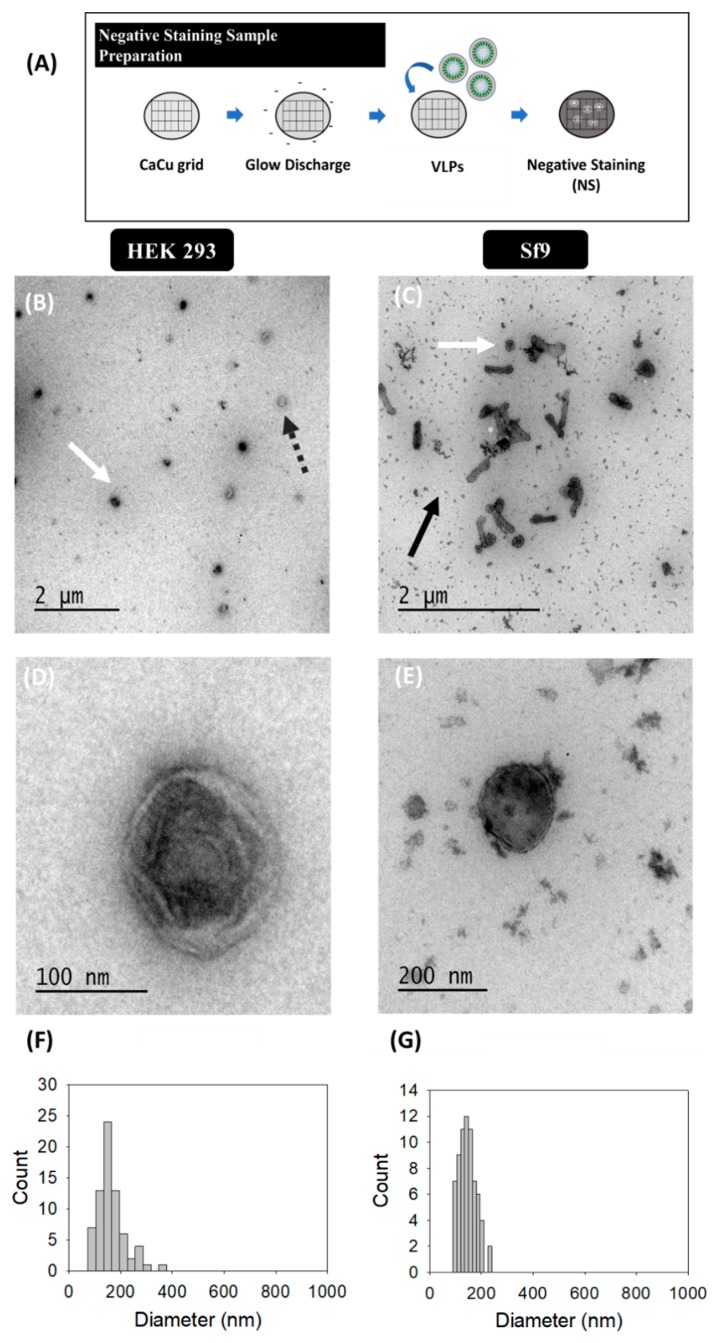
Transmission electron microscopy with negative staining analysis of HIV-1 Gag-eGFP VLPs produced in HEK 293 and Sf9 cells harvested at 72 hpt and 72 hpi, respectively. (**A**) Sample preparation protocol; (**B**–**E**) samples corresponding to the 25–45% ultracentrifugation fraction from HEK 293 supernatants (**B**,**D**) and Sf9 supernatants (**C**,**E**). All samples were stained with uranyl acetate in Ca-Cu grids. (**F**,**G**) PSD analysis of HEK 293 and Sf9 concentrated samples, respectively. White and grey dashed arrows indicate the presence of VLPs and EVs in HEK 293 and Sf9 productions, respectively, and black arrows indicate the presence of BVs in Sf9 samples. Negative controls were assessed using the same conditions as in VLP samples (Supplementary materials S1).

Prior to negative staining, HIV-1 Gag-eGFP VLPs were concentrated by double sucrose cushion 25–45% (w:v) at 31.000 rpm and centrifuged for 2.5 h at 4 °C in a Beckman Optima L100XP centrifuge using a SW32 Ti rotor (Beckman Coulter, Brea, CA, USA). The 25–45% interphase was recovered and stored at 4 °C until analysis [41]. TEM micrographs were analyzed after air-dried negative staining. The protocol used is summarized in Figure 1A. Briefly, VLP samples were deposited onto carbon-coated copper or Holey carbon 200 mesh grids (Micro to Nano, Wateringweg, the Netherlands). Grids were glow discharged in a PELCO easiGlow glow discharge unit (PELCO, Fresno, CA, USA). Thereafter, 8 μL of sample were loaded onto the grid and incubated at RT for 1 min. Excess sample was carefully drained off the grid with the aid of filter paper. Samples were negatively stained with 8 μL of 2% w:v uranyl acetate by incubation at RT for 1 min. Excess stain was drained off as previously indicated and grids were dried at RT until analysis. TEM examinations were performed with a Jeol JEM-1400 (JEOL USA, Pleasanton, CA, USA) transmission electron microscope equipped with an ES1000W Erlangshen charge-coupled device camera (CCD) (Model No. 785; Gatan, Pleasanton, CA, USA).

#### 2.3.2. Scanning Electron Microscopy (SEM)-Alcian Blue Staining

**Figure 2:**
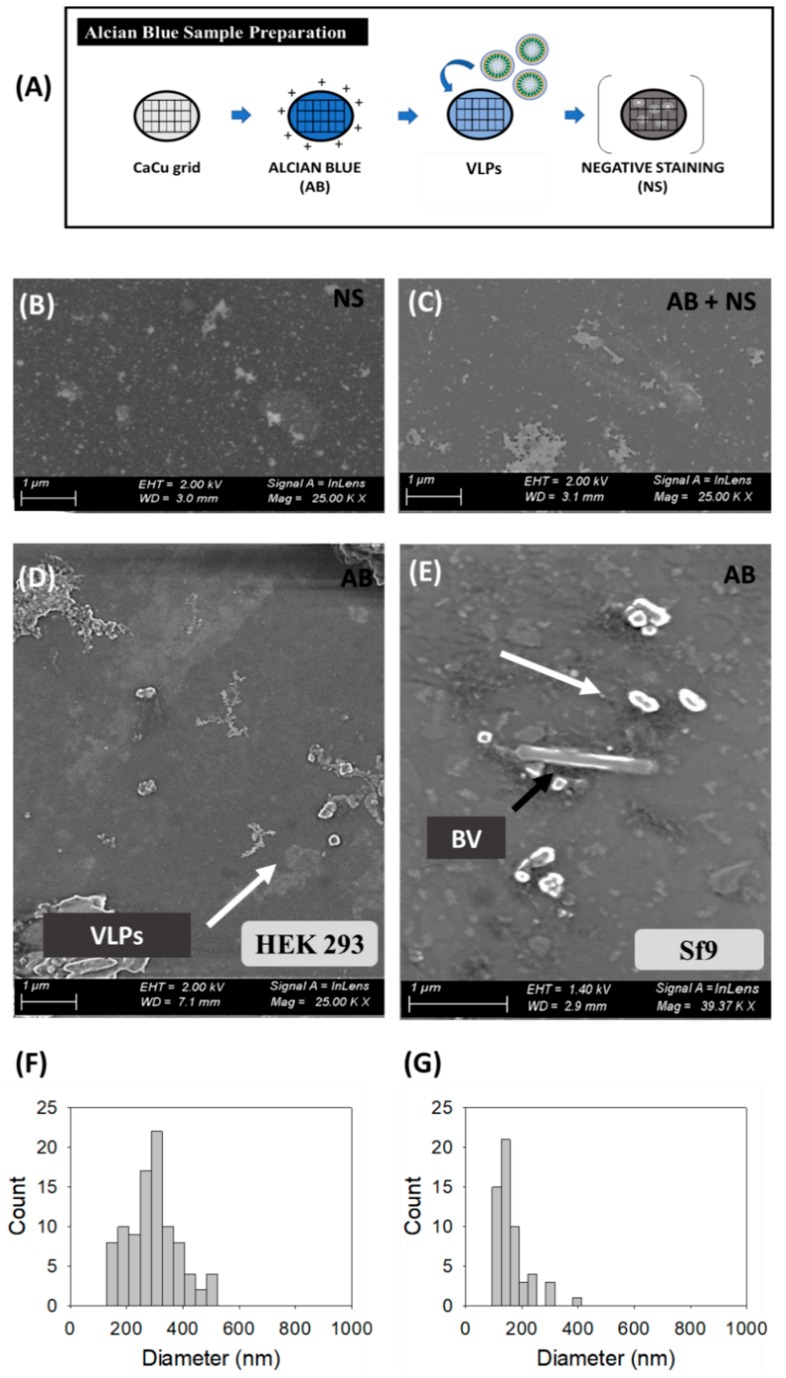
Scanning electron microscopy analysis of HIV-1 Gag-eGFP VLPs produced in HEK 293 and Sf9 cell lines harvested at 72 hpt and 72 hpi, respectively. (**A**) Sample preparation protocol; (**B**–**E**) Comparison of different sample preparation methods: (**B**) negative staining (NS) (**C**), combining Alcian Blue solution and negative staining (AB + NS) on HEK 293 supernatants; (**D**) Supernatant from transfected HEK 293 cells and (**E**) supernatant from BV infected Sf9 cells treated with Alcian Blue (AB) for 1 min in a Holey carbon 200 mesh grid. (**F**–**G**) PSD analysis of HEK 293 and Sf9 supernatants, respectively. White arrows indicate the presence of nanoparticles in HEK 293 and Sf9 supernatants and black arrows indicate the presence of BV. Negative controls were analyzed using the same conditions as in VLP samples (Supplementary materials S1).

HIV-1 Gag-eGFP VLP visualization by SEM was performed by staining VLP-containing supernatants with Alcian Blue solution, adapted from Gállego [17]. The protocol used is shown in Figure 2A. Briefly, Alcian Blue solution 1% w:v in 3% v:v acetic acid (Sigma Aldrich, St Louis, MO, USA) was diluted in ultrapure water achieving a final concentration of 1 µg/mL. Carbon-coated copper or Holey carbon 200 mesh grids were placed in this solution for 5 min, then the excess of Alcian Blue solution was removed by washing the grid in ultrapure water. Grids were dried with filter paper. Thereafter, 8 μL of the sample were placed on the grid and incubated at RT for 5 min. Excess sample was carefully drained off the grid with the aid of filter paper. Negative staining was applied to the samples when required, using the protocol described in Section 2.3.1. SEM micrographs were performed in a FE-SEM Merlin scanning electron microscope (Zeiss, Jena, Germany). Micrographs were taken with the lens mode and secondary electron detector, with an electron high tension (EHT) comprised between 1–2 eV and 2.9–7.5 mm of working distance using a protocol adapted from González-Domínguez et al. [31].

#### 2.3.3. Cryogenic Transmission Electron Microscopy (cryo-TEM)

**Figure 3:**
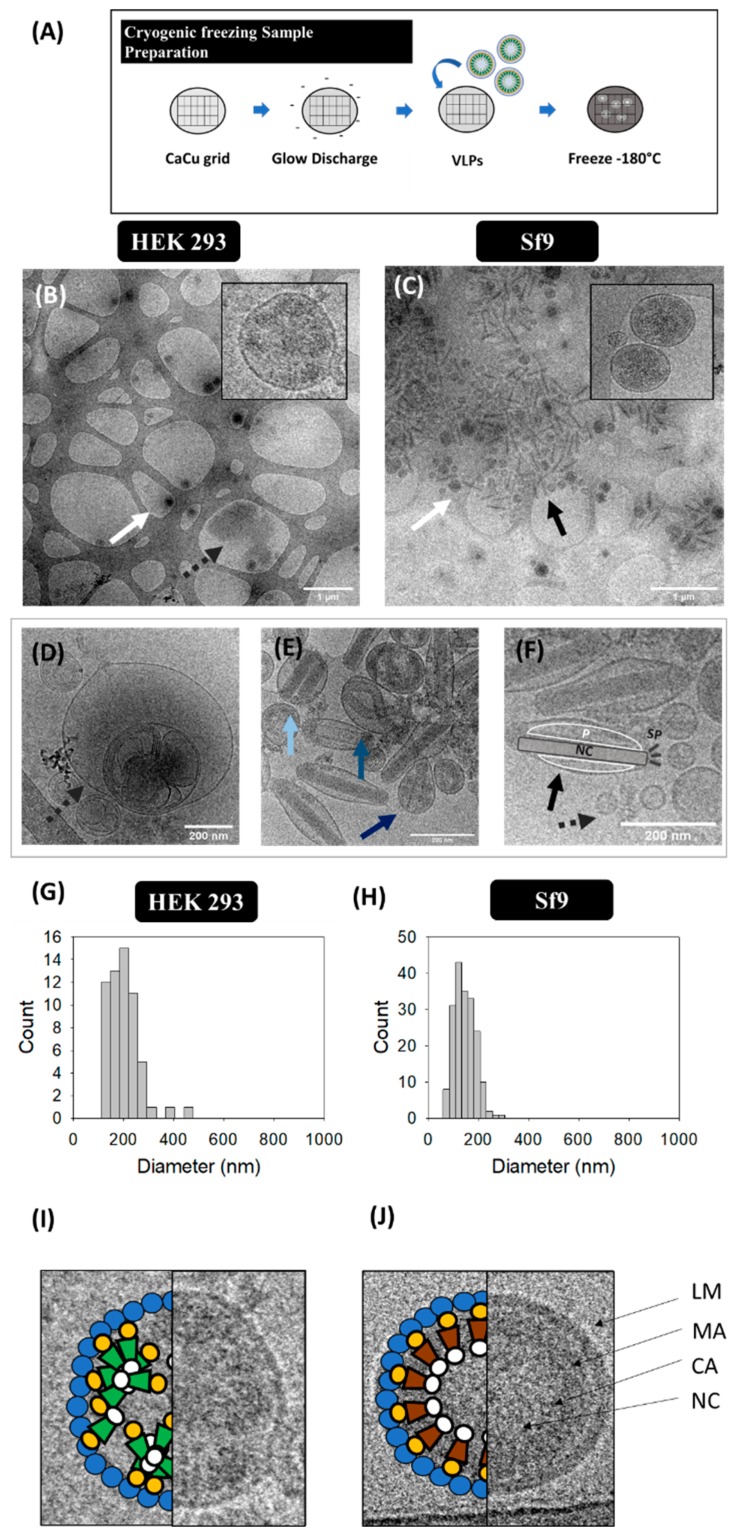
Cryo-TEM analysis of HIV-1 Gag-eGFP VLPs produced in HEK 293 and Sf9 cells harvested at 72 hpt and 72 hpi, respectively. (**A**) Sample preparation protocol. Supernatant from HEK 293 (**B**) and Sf9 cells (**C**). Both samples were prepared and visualized in Holey carbon grids. (**D**–**F**) Morphological characterization of contaminant particles including multivesicular bodies (MVB) (**D**), occlusion-derived BV (ODV, light blue), a relaxed-form of the BV (rBV, mid blue) or BV-containing vesicles or protein structures (cBV, dark blue) (**E**), and analysis of the infective BV (budded virus) structure: nucleocapsid (NC), lateral pocket side (P) and apical spikes (SP) (**F**). (**G**–**H**) PSD analysis of HEK 293 and Sf9 supernatants, respectively. White arrows indicate the presence of VLPs, dashed grey arrows point EVs and infective BVs are shown in black arrows. (**I**,**J**) Ultrastructural organization of an HIV-1 Gag-eGFP VLP (**I**) and an HIV-1 Gag VLP (**J**) produced in Sf9 cells by BV infection. LM: lipid membrane, MA: matrix; CA: capsid, NC: nucleocapsid. Negative controls were assessed using the same conditions as in VLP samples (Supplementary materials S1).

Cryo-TEM analyses of HIV-1 Gag-eGFP VLPs were conducted from harvested supernatants. In Figure 3A, the sample preparation procedure for VLP visualization is presented. 2–3 μL of sample were blotted onto 200 or 400 mesh Holey carbon grids (Micro to Nano, Wateringweg, the Netherlands) previously glow discharged in a PELCO easiGlow glow discharge unit. Samples were subsequently plunged into liquid ethane at −180 °C using a Leica EM GP cryo workstation and observed in a Jeol JEM-2011 TEM electron microscope operating at 200 kV. During imaging, samples were maintained at −173 °C, and pictures were taken using a CCD multiscan camera (Model No. 895, Gatan).

### 2.4. Super-Resolution Fluorescence Microscopy (SRFM)

**Figure 4:**
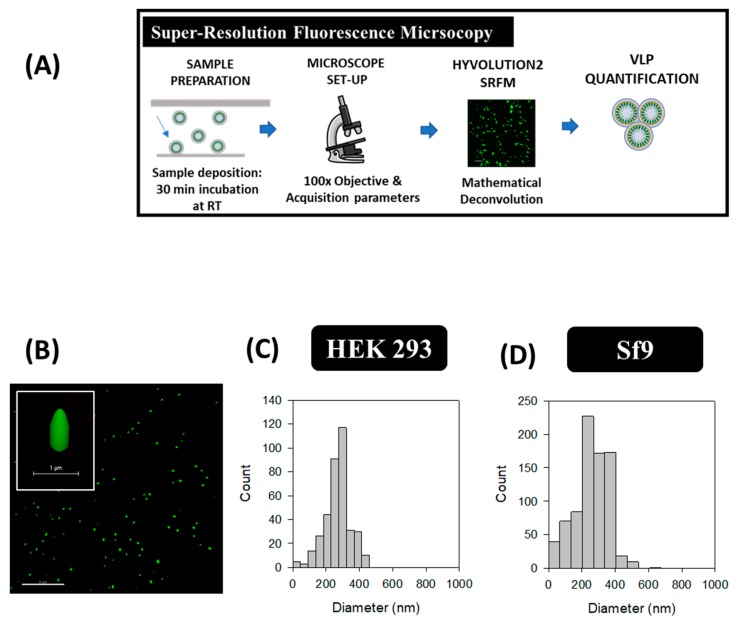
Super-resolution fluorescence microscopy analysis of HIV-1 Gag-eGFP VLPs produced in HEK 293 and Sf9 cells harvested at 72 hpt and 40 hpi, respectively. (**A**) Sample preparation and analysis workflow and (**B**) Gag-eGFP VLP imaging. (**C**,**D**) PSD analysis of HEK 293 and Sf9 supernatants directly quantified after their addition to the microscope slide, respectively. Negative controls were analyzed in the same conditions as in VLP samples (Supplementary materials S1).

SRFM was performed with a TCS SP8 confocal microscope equipped with Huygens deconvolution suite embedded via a direct interface with LAS X software and GPU arrays (Leica Microsystems, Wetzlar, Germany) at Servei d’Anatomia Patològica from Hospital Sant Joan de Déu (Esplugues de Llobregat, Barcelona, Spain), as previously described [24]. A summary of the protocol is depicted in Figure 4A. Briefly, harvested VLPs were directly loaded onto the microscope slide and adsorbed to the surface of the cover glass after an incubation time of 30 min at RT (Figure 4A). HIV-1 Gag-eGFP VLP preparations were analyzed with 100 X magnification (zoom 5), a line average of 3 and 496 x 496 pixels with HC PL APO CS2 100 X/1.40 OIL objective with the HyVolution2 mode (Leica). Five fields of 13 sections per each biological triplicate were studied in harvested HIV-1 Gag-eGFP VLPs. Deconvolution was performed with the SVI Huygens Professional program and the best resolution strategy (Scientific Volume Imaging B.V., Hilversum, the Netherlands). HIV-1 Gag-eGFP VLP concentration was calculated based on the division of particle number by 3D image volume as previously described [24]. Briefly, direct quantification was performed on deposited HIV-1 Gag-eGFP VLP samples with 23 × 23 × 3 µm in *xyz* from a total loaded volume of 50 µL distributed in 24 × 60 mm under the cover glass and a total height of 34 µm. Assuming complete sample deposition, minimum concentration of Gag-eGFP VLPs was also calculated. PSD analyses were performed using SigmaPlot 12.0 software.

### 2.5. Nanoparticle Tracking Analysis (NTA)

**Figure 5:**
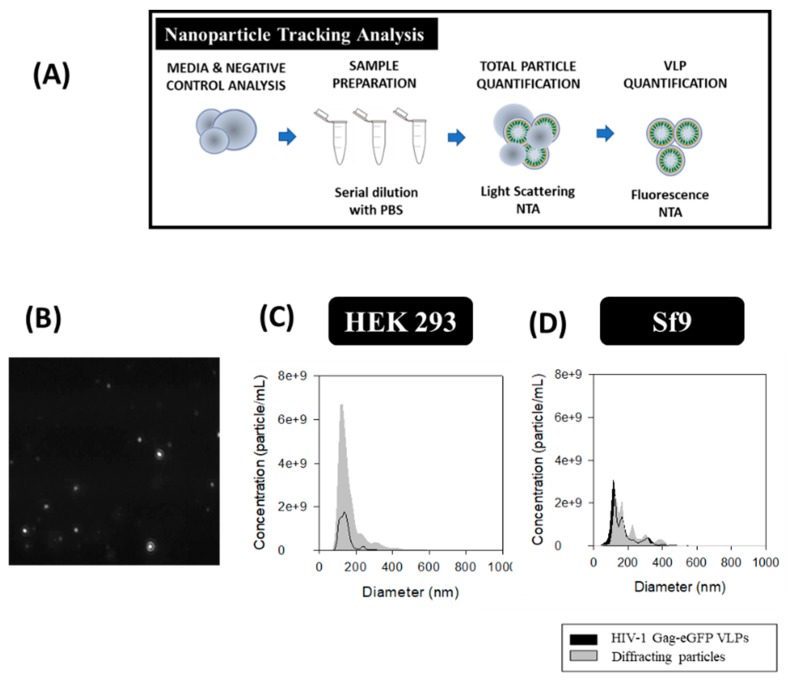
Nanoparticle tracking analysis of HIV-1 Gag-eGFP VLPs produced in HEK 293 and Sf9 cells harvested at 72 hpt and 40 hpi, respectively. (**A**) Sample preparation and analysis workflow. (**B**) Image of nanoparticles tracked by NTA. (**C**,**D**) PSD analysis of HEK 293 and Sf9 supernatants, respectively. HEK 293 and Sf9 supernatants were diluted with filtered DPBS to adjust their concentration prior to analysis. Negative controls were analyzed in the same conditions as in VLP samples (Supplementary materials S1).

HIV-1 Gag-eGFP VLPs and total particle content were analyzed by NTA. A NanoSight^®^ NS300 device (Malvern Panalytical, Malvern, United Kingdom) equipped with a blue filter module (488 nm) and a neutral filter was used to quantify GFP-fluorescent nanoparticles and total particle by light diffraction, respectively. The measurements were performed at Service of Preparation and Characterization of Soft Materials located at Institut de Ciència de Materials de Barcelona (ICMAB, CSIC, Campus UAB). The workflow used for HIV-1 Gag-eGFP VLP quantification is summarized in Figure 5A. Prior to injection into the device chamber, each sample was diluted to obtain 1 mL sample with a concentration of around 10^8^ particles/mL. Sample injection into the chamber was continuously added using a pump to improve the robustness of the measurement and minimize the photobleaching effect due to fluorescence depletion over time (Figure 5B) [42]. The videos recorded were then analyzed with the NTA 3.2 software (Malvern Panalytical) by tracking the individual particle movement, where camera level and detection threshold were adjusted manually for each sample (Table 1). Three independent analyses were carried out and videos of 60 s were recorded at RT, with particles identified and tracked by their Brownian motion. HIV-1 Gag-eGFP VLP concentrations were calculated as the total fluorescent particles and the concentration of EVs was calculated as the difference between light scattering particles and fluorescent particles. PSD analyses were performed with NTA 3.2 software and SigmaPlot 12.0 software.

### 2.6. Flow Virometry

**Figure 6:**
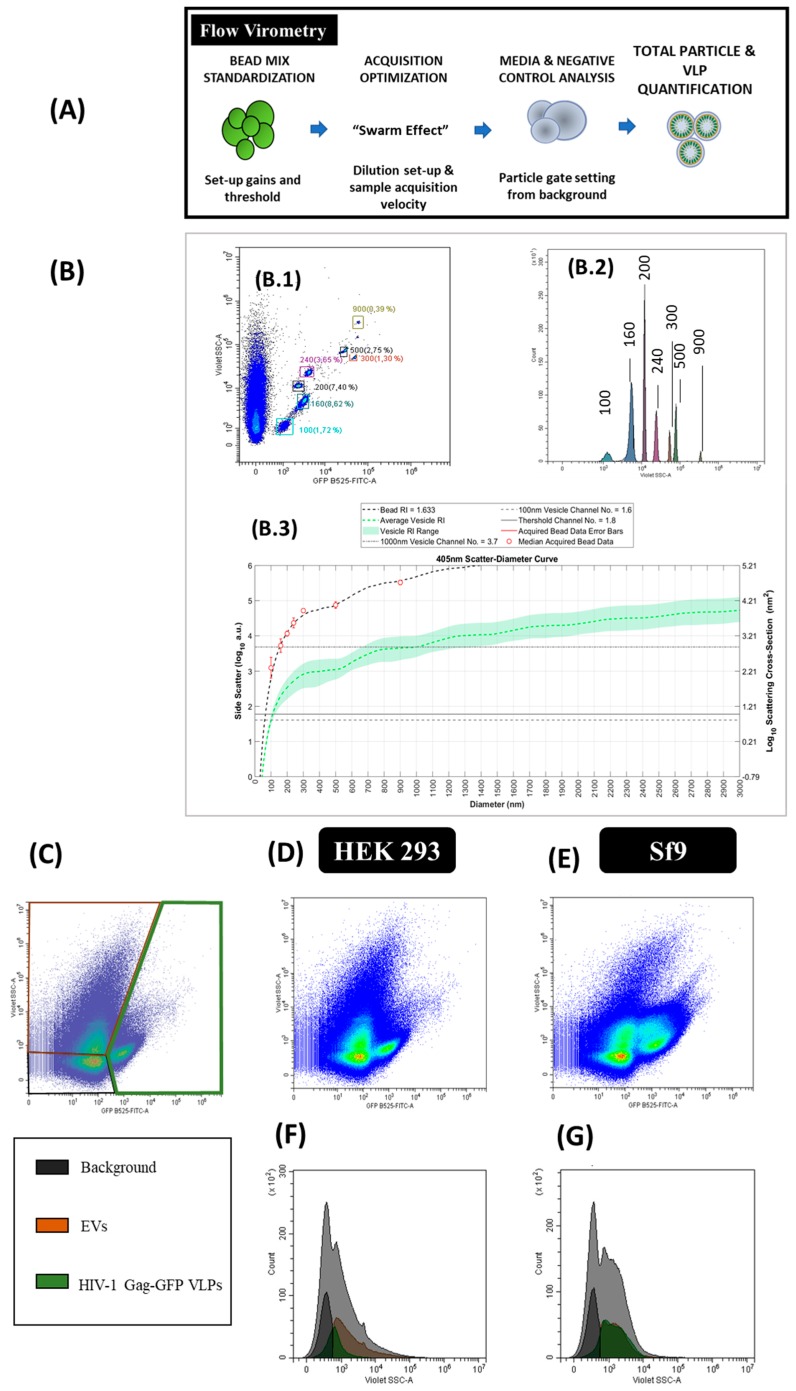
Flow virometry analysis of HIV-1 Gag-eGFP VLPs produced in HEK 293 and Sf9 cells harvested at 72 hpt and 40 hpi, respectively. (**A**) Sample preparation and analysis workflow and (**B**) equipment calibration using beads with a known diameter. Megamix-Plus fluorescent beads of 100, 160, 200, 240, 300, 500 and 900 nm were used. (B.1) GFP B525-FITC-A vs. V-SSC density plots of the beads, (B.2) GFP B525-FITC-A histogram of the beads and (B.3) Mie correlation of the beads with FCM_PASS_ software [33]. (**C**) Gating of the different nanoparticle populations in a density plot and defined as EVs, HIV-1 Gag-eGFP VLPs and background signal of the equipment. (**D**–**G**) Density plots and histograms of nanoparticles produced in HEK 293 (**D**,**F**) and Sf9 supernatants (**E**,**G**), respectively. HEK 293 and Sf9 supernatants were diluted with filtered DPBS prior to analysis. Negative controls were analyzed using the same conditions as in VLP samples (Supplementary materials S1).

Flow virometry experiments were performed with a CytoFLEX LX (Beckman Coulter) with violet side scatter (V-SSC) 405 nm filter configuration at Servei de Cultius Cel·lulars, Producció d’Anticossos i Citometria (UAB, Barcelona, Spain). The different steps required in the analysis are described in Figure 6A. Measurements from different experiments were standardized using a mixture of Megamix-Plus Side Scatter and Forward Scatter (FSC) fluorescent polystyrene beads (100, 160, 200, 240, 300, 500 and 900 nm; Biocytex, Marseille, France). The threshold of height trigger signal in Violet Side Scatter (V-SSC) was manually adjusted to 1200 and laser gains were set as 72, 9 and 106 for FSC, V-SSC and B525-FITC, respectively. Samples were diluted with 1 X Dulbecco’s phosphate-buffered saline (DPBS, Thermo Fisher Scientific) until the abort rate value was below the 2%. Three-hundred thousand events were analyzed per sample at a flow rate of 10 µL/min. V-SSC vs B525-FITC density plots were used to gate the different EVs and VLPs populations. Gating was manually adjusted for each channel. Results were analyzed with the CytExpert v.2.3 software (Beckman Coulter). Nanoparticle concentrations were calculated with Equation (1):

(1) $$Particle\;concentration\;\left(\frac{Events}{mL}\right)=\left(Events\right)\cdot \frac{µL}{\mathrm{mL}}\cdot Dilution$$

Particle size diameters based on the median violet side scatter were calculated by Mie correlation [43] using FCM_PASS_ software [33]. Megamix-Plus SSC and FSC fluorescent polystyrene beads were used as the reference material with a refractive index (RI) of 1.633 with a 405 nm illumination wavelength [43]. The following RI were used for Mie correlation: 1.374 (vesicle cytosol), 1.394 (vesicle upper cytosol), 1.354 (vesicle lower cytosol), 1.474 (vesicle membrane) and 1.345 (vesicle surrounding medium) considering 405 nm as the illumination wavelength. The vesicle membrane thickness was defined as 10 nm [43]. The half angle parameter was set as circular aperture geometry. The maximum diameter was fixed as 3000 nm, the triggering threshold as 60 arbitrary units (a.u.) and the log decade number parameter comprised between 0–6. Thereafter, HIV-1 Gag-eGFP VLP and EV mean sizes were exported with the FlowJo v.10 software (BD Biosciences).

## 3. Results

### 3.1. Transmission Electron Microscopy (TEM)-Negative Staining

Virus-like particle (VLP) characterization by TEM with uranyl acetate as the negative staining solution is shown in Figure 1. VLPs were concentrated by ultracentrifugation according to previous studies [36]. VLPs produced in HEK 293 (Figure 1B,D) and Sf9 cells (Figure 1C,E) were examined in order to identify differences arising from the expression of these nanoparticles in each specific cell platform. Uranium strongly reacts with phosphate and amino groups and typically stains proteins, nucleic acids and lipid membranes [44]. Thus, VLPs were observed as spherical electrodense structures surrounded by a bright corona (white arrows) that might correspond to structured Gag-eGFP monomers surrounded by the lipid membrane [36,45]. As expected, the presence of baculoviruses (BV) (black arrows) was detected along with VLPs (white arrows) in infected Sf9 samples. EVs were observed as less electrodense nanoparticles (dashed grey arrows) and that could also be identified in conditioned medium samples (Supplementary materials S1) [46]. Interestingly, differences between VLPs and EVs were less evident in Sf9 micrographs compared to HEK 293 ones. A mean population diameter of 165 ± 54 nm (*n* = 71) and 146 ± 33 nm (*n* = 69) was quantified in HEK 293 and Sf9 samples, respectively (Figure 1F,G). Moreover, TEM characterizations revealed that only the 52 ± 6% and 55 ± 14% of the nanoparticles analyzed were VLPs in HEK 293 and Sf9 concentrated samples. The use of negative staining for sample visualization hindered a more detailed characterization of the structure of the different specimens due to the presence of artifacts and background noise in all samples (Figure 1B,E) [1].

### 3.2. Scanning Electron Microscopy (SEM)-Alcian Blue Staining

SEM analyses were directly performed on harvested VLP supernatants from transfected HEK 293 (Figure 2B,D,F) and BV infected Sf9 cells (Figure 2E,G). Initial analyses using standard sample preparation protocols resulted in the loss of a high portion of the nanoparticles prior to visualization [47]. Thus, the combination of SEM with Alcian Blue solution was investigated (Figure 2) and compared to uranyl acetate staining as the reference methodology. The comparison between negative staining, Alcian Blue grid pre-treatment followed by negative staining, and only Alcian Blue grid pre-treatment is shown in Figure 2B–E, respectively.

Samples stained with uranyl acetate presented an important background regardless the grid treatment or not with Alcian Blue solution (Figure 2B,C and Figure S1). Indeed, large and irregular salt stacks deposited on the sample grid were detected probably related to the interaction of uranium with phosphate salts and amino acids from cell culture media, with similar results as observed in TEM micrographs (Figure 1). A decrease in the load of background signal was achieved by Alcian Blue grid pre-treatment without negative staining (white arrows) and nanoparticles could be individually resolved as 3D sphere-like structures. Despite the improvement in nanoparticle resolution achieved by the Alcian Blue grid pre-treatment, VLPs and EVs could not be distinguished in these analyses. Thus, the calculation of particle size distribution (PSD) was performed considering all nanoparticles as a single population, resulting in 296 ± 88 nm (*n* = 94) for HEK 293 and 162 ± 60 nm (*n* = 57) for Sf9 supernatants. The presence of EVs in conditioned supernatants could also be observed by SEM-Alcian Blue staining (Supplementary Materials S1), as well as the typical rod-shaped capsids of BVs (black arrows) in infected Sf9 supernatants (Figure 2E).

### 3.3. Cryogenic Transmission Electron Microscopy (cryo-TEM)

Ultrastructural analysis of VLP, EV and BV populations was conducted in harvested supernatants by cryo-TEM (Figure 3B–F). Gag-eGFP VLPs were observed as electrodense nanoparticles surrounded by a lipid envelope with a granular-like heterogeneous internal structure (Figure 3B,C, white arrows). Gag-eGFP VLPs produced in both platforms displayed an average size of 202 ± 68 nm (*n* = 59) for HEK 293 and 146 ± 42 nm (*n* = 188) for Sf9 cells (Figure 3G,H, respectively). In parallel, different EV subpopulations could be detected, including exosomes (30–100 nm), microvesicles (50–2000 nm) and multivesicular bodies (MVB) (Figure 3D–F) [6]. Similar nanoparticle populations were detected in conditioned medium samples from both cell lines (Supplementary materials S1), indicating a basal expression of EVs in these cell lines. As for infected Sf9 supernatants, a large concentration of BVs was detected (black arrows), encompassing different BV phenotypes: the occlusion-derived BV (ODV), a relaxed form of the BV (rBV) (Figure 3E) and the typical infective BV (budded virus) (Figure 3F). ODVs presented several rod-shaped nucleocapsids arranged in parallel disposition inside vesicular bodies (light blue arrow), while a spiral-like nucleocapsid organization could be clearly distinguished in rBV (mid blue arrow) [7]. Budded BVs displayed ovoid-like structures containing one nucleocapsid, with the DNA highly compacted in supercoiled structures (black arrow). Moreover, the apical spikes and the BV lateral pocket, which is the space between the lipid bilayer and the nucleocapsid, were resolved using this technique (Figure 3G) [7]. Other BV forms could be identified in infected Sf9 samples and consisted of BV containing vesicles or protein structures besides the nucleocapsid (cBV, dark blue arrow, Figure 3E). Differences in Gag-eGFP VLPs (green) and Gag VLPs (brown) were also evaluated by cryo-TEM (Figure 3I,J). Gag VLPs evidenced a higher internal degree of ordered Gag arrangement in comparison to Gag-eGFP VLPs, similar to that of immature HIV-1 virions [38], while Gag-eGFP VLPs did not attain such level of structural organization.

### 3.4. Super-Resolution Fluorescence Microscopy (SRFM)

HyVolution2 SRFM nanoparticle analyses were conducted in HEK 293 and Sf9 supernatants by a simple preparation strategy similar to common cell visualization methods used in confocal microscopy. Images were acquired at 100 X magnification as depicted in Figure 4B, where VLPs correspond to green dots. VLPs presented an elongated structure in the Z plane while a smaller diameter was observed in the XY axis due to the convolution effect of light. A mean VLP diameter of 268 ± 77 nm (*n* = 371) was obtained in HEK 293 samples, whereas an average of 261 ± 104 nm (*n* = 794) was measured for Sf9 supernatants (Figure 4C,D). VLP concentrations were comprised in the range of 10^9^–10^10^ VLPs/mL in both platforms, representing a 1.7-fold difference for Sf9 over HEK 293-derived VLPs (Table 2). The same acquisition strategy was performed in conditioned medium samples from both cell lines, and no detection of GFP signal was observed (Supplementary materials S1).

### 3.5. Nanoparticle Tracking Analysis (NTA)

The total amount of nanoparticles produced in each platform was evaluated by NTA with the light scattering mode and VLPs were analyzed with the fluorescent filter module (Figure 5A). The PSD and concentration of the different nanoparticle populations was conducted by the combination of both modules. An average diameter of 143 ± 39 nm for VLPs and 161 ± 66 nm considering all nanoparticles as a single population was measured in HEK 293 samples. As for Sf9 supernatants, an average diameter of 213 ± 95 nm was measured for VLPs and 194 ± 75 nm for total nanoparticles. Comparison of VLP concentrations in both platforms resulted in a 1.7-fold increase in Sf9 compared to HEK 293 samples (Figure 5C,D). On the contrary, a higher EV content was found in HEK 293 samples (Table 2). Analysis of original FreeStyle and Sf900III cell culture media by light scattering NTA resulted in a concentration of 0.6 ± 0.1 × 10^9^ and 24.4 ± 1.1 × 10^9^ diffracting particles/mL, respectively, with a mean size diameter of 174 ± 39 nm and 92.7 ± 74.6 nm. Thus, a difference of two orders of magnitude in particle concentration was obtained in Sf900III over FreeStyle medium. Evaluation of conditioned FreeStyle and Sf900III media was also assessed by NTA and yielded a concentration of 17.6 ± 0.9 × 10^9^ and 27.3 ± 1.5 × 10^9^ diffracting particles/ml, respectively, with a mean diameter of 174 ± 65 nm and 101 ± 47 nm for HEK 293 and Sf9 samples, respectively.

### 3.6. Flow Virometry

The production of HIV-1 Gag-eGFP VLPs and other nanoparticle populations was simultaneously analyzed by flow virometry. Equipment calibration for VLP analysis was performed with commercial beads of known diameter (100, 160, 200, 240, 300, 500 and 900 nm) in order to calculate the mean nanoparticle diameter (Figure 6B, B.1 and B.2). Mie scatter modelling allowed to convert V-SSC intensities of VLPs and EVs to their corresponding size with FCM_PASS_ software developed by Welsh and co-authors (Figure 3 and Figure 6 and Supplementary materials S2) [33]. By doing so, the detection of nanoparticles with diameters down to 100 nm could be achieved.

After equipment set-up, different nanoparticle populations could be assessed in one single analysis. Three main populations were detected in transfected HEK 293 and BV infected Sf9 samples (Figure 6G). Two of these populations corresponded to EVs, classified as small and large EVs, and the third one was related to VLPs (fluorescent particles). EVs displayed a high level of heterogeneity in the V-SSC, with values ranging from 10^2^ to 10^5^ a.u. Interestingly, a second subpopulation of VLPs with a higher V-SSC intensity was detected in BV infected Sf9 samples, which could be probably associated to the aggregation of VLPs or the interaction of VLPs with other cellular compounds released to the medium. This second VLP subpopulation displayed a more pronounced right-skewed V-SSC distribution compared to the more homogeneous VLP population observed in HEK 293 samples (Figure 6F). In terms of quantification, VLP concentrations in the range of 10^8^ particles/mL were measured with a 1.5-fold increase in VLP concentration in Sf9 over HEK 293 supernatants (Table 2). As regards EVs, higher levels of these nanoparticles were quantified in conditioned media from Sf9 over HEK 293 cells, respectively (Supplementary materials S1). Mean size analysis of VLPs and EVs by Mie correlation resulted in 114 ± 26 nm for VLPs and 115 ± 26 nm for EVs in HEK 293 supernatants, respectively, while a mean diameter of 117 ± 22 nm and 115 ± 24 nm was measured for VLPs and EVs in Sf9 supernatants (Figure 6D–G).

## 4. Discussion

### 4.1. Sample Preparation and Equipment Set-up

Among the different techniques used, the cost, technical requirements and the time needed for sample analysis are practical issues that have to be considered to select an adequate analytical technique for nanoparticle characterization (Table 3) [12]. Regarding sample preparation, purified VLPs were loaded and stained by the addition of uranyl acetate in TEM-Negative staining. Altogether, sample preparation time required less than 10 min per sample, without considering the previous ultracentrifugation step, which required one day of experimentation. Compared to the TEM-Negative staining protocol (Figure 1A), SEM-Alcian Blue grids were not treated by glow discharge before the addition of Alcian Blue solution. The VLP-containing supernatant was directly loaded, representing an overall process duration of 15 min. As for cryo-TEM, sample preparation required an approximate time of 10 min; however, the equipment set-up required a longer time of pre-conditioning compared to TEM and SEM due to the low temperature conditions employed. In SRFM, acquisition of 3D VLP images took around 10 min per field and data analysis with Imaris software required 15–20 min to process the different channels and construct the final image. As for NTA, the time needed to conduct a complete analysis lasted 1 h due to sample preparation and software analysis (Figure 5A), while in flow virometry the different nanoparticle populations could be analyzed in one single run (Table 3). 

In most cases, sample preparation and equipment set-up are based on trial-and-error, which can introduce a certain degree of variability in the characterization of nanoparticles. In the case of TEM, the low electron density present in biological samples as well as the need to work under vacuum conditions require the addition of contrast agents for a better resolution of the specimens under evaluation. Uranium strongly reacts with phosphate and amino groups [44], thus conferring a higher level of electron density to biological samples and VLPs are observed as round structures surrounded by a bright corona. Despite the simplicity of negative staining, the presence of artifacts and background due to the composition of the contrast medium are detected (Figure 1B–D), as reported in the literature [3,15]. Depending on the sample origin, this can have a major impact, as observed in Sf9 cell micrographs in comparison to HEK 293 samples, where a higher level of background was identified and EVs could not be distinguished from VLPs (Figure 1C). This background is probably caused by the larger load of particles present in insect cell culture media [49]. In this sense, new commercially available chemically defined insect cell culture media could alleviate this problem [50]. Different approaches have been investigated to overcome the drawbacks of negative staining [1]. SEM-Alcian Blue has been studied in this work as a possible alternative. On the one hand, TEM has been traditionally used for viral intraparticle characterization, while SEM is generally applied for a broader screening of biological populations [48,51]. Novel preparation methods, such as ionic liquid infiltration [51] or virus quantification by Prep/g [52] have been reported as strategies to assess bacterial and viral preparations with SEM in the recent years. In the same line, the use of Alcian Blue as a solution for grid pre-treatment was shown to increase the adsorption of nanoparticles in this work, thus allowing to improve the detection of these particles avoiding negative staining (Figure 2). Nevertheless, the differentiation of VLPs from EVs was not possible due their similar morphology, but this new label-free method has a potential applicability in combination with X-ray spectroscopy [31], SRFM [53], or “wet” SEM [51] to deepen into nanoparticle characterization. Alternatively, the low contrast present in biological samples can also be overcome in EM by using cryo-TEM, which does not require sample staining (Figure 3). However, an adjustment of the cryogenic freezing protocol for each sample depending on its physicochemical properties is required [21]. Overall, all EM techniques demand a high expertise by the user and often entail long sample analysis times (Table 3). Therefore, the development of more automatic and high throughput complementary methods is required to process nanoparticle samples in a faster manner.

SRFM, NTA and flow virometry can be implemented to this purpose since they allow mass quantification of nanoparticles. Nonetheless, sample standardization and technical expertise is still critical for an adequate nanoparticle assessment (Table 3). A microscope equipped with advanced capture and image processing modules, and the optimization of acquisition conditions together with different imaging software are required in SRFM. Additional process automatization should be developed to widen its application since the time required for sample analysis is still high and operator-dependent. In NTA, relevant parameters to be considered during nanoparticle analysis are the camera level and the detection threshold [5]. These settings need to be manually adjusted in each sample for an adequate tracking of the nanoparticles recorded and their automatic quantification. NTA measurements depend on the refractive index (RI) of each particle and the nanoparticle containing solution. Thus, different specific settings were applied to measure HEK 293 and Sf9 supernatants (Table 1). Another important feature affecting the final output with NTA is size heterogeneity of nanoparticle populations in the sample, which hinder the quantification of the small subpopulations due to the screening effect of higher particles, as reported by van der Pol et al. [5]. Regarding flow virometry, differences in the measurements due to inter-equipment variability have been pointed as one of the major challenges of this technique. Nevertheless, the use of commercial beads as standard nanoparticles and the implementation of Mie correlations have contributed to solve this problem [33,52,54]. Similarly, tunable resistive pulse sensing (TRPS), Atomic Force Microscopy (AFM) and field-flow fractionation coupled to multiangle light scattering (FFF-MALS) are single particle analysis methods that can be implemented for nanoparticle characterization [12]. However, none of these techniques enable to differentiate VLPs from EVs without additional sample treatments.

### 4.2. Ultrastructural Analysis

Single particle evaluation conducted by SRFM, NTA and flow virometry is an interesting option for characterization analyses since specific labeling can be applied to study different nanoparticle populations [27,55]. The use of the fluorescently tagged Gag polyprotein enabled the differentiation of VLPs from the other co-produced nanoparticle populations in this work. These analytical methods have also been successfully used in combination with immunolabeling to quantitatively asses viral populations [27]. A step further has been recently achieved in SRFM with the possibility to detect nucleic acids and the lipid membrane in VLPs [25]. The commercially available ViroCyt® flow cytometer has been similarly used to quantify different viral isolates, including the Ebola virus [56]. Still, the detection of VLPs with ViroCyt^®^ has not been reported [57]. Nevertheless, none of these techniques achieve the levels of nanoparticle resolution of EM methods (Table 3).

Among the different EM methods evaluated, cryo-TEM showed a higher benefit compared to TEM and SEM since the native conformation of the different nanoparticles could be assessed. The ultrastructural analysis of the different VLP, EV and BV subpopulations could be described in detail, with remarkable differences detected between Gag-eGFP and Gag VLPs. The Gag polyprotein is known to travel to the vicinity of the plasma membrane, aggregate with other Gag monomers, and bud to the extracellular space as an immature HIV-1 particle (Figure 3J) [38,58]. Interestingly, Gag-eGFP VLPs do not achieve the expected organized structure as Gag VLPs probably due to the eGFP fusion that alters the native budding process [59]. These differences could not be detected by TEM due to the interference of negative staining with the samples [11,36,60]. To our knowledge, this is the first time were Gag-eGFP VLP intraparticle organization is observed. Therefore, it is shown that fusion proteins can introduce morphological alterations in the structure of the nanoparticles produced and cryo-TEM is an interesting method to identify them.

### 4.3. Particle Size Distribution

The PSD of VLPs has been studied with the six methods evaluated in this work. Typically, the VLP mean diameter is comprised between 100 and 200 nm, as observed by TEM, cryo-TEM, NTA and flow virometry [61]. Non-symmetric right-skewed distributions were observed for VLPs measured with the different techniques. Similar PSDs of HIV-1 Gag VLPs produced in CAP-T and CHO cells were shown by Gutierrez-Granados et al. and Steppert et al., respectively [37,62]. The presence of larger population of VLPs, especially when measuring Sf9 samples by NTA and flow virometry, could be related to the aggregation of VLPs or their interaction with other cellular compounds released to the medium. SEM and SRFM presented higher PSD in comparison to the rest of analytical technologies. SEM analysis of HEK 293 samples resulted in a PSD of 296 ± 88 nm, which could indicate that several EVs were quantified instead of VLPs since microvesicles can reach up to 2000 nm [6]. HyVolution2 SRFM yielded the highest VLP PSD values in both cell platforms (>250 nm), which has also been observed by Xiao and co-workers when analyzing Cy5-labeled adeno-associated viruses [63], and could be attributed to the convolution effect of light. In this case, the individual fluorochrome intensity does not necessarily correlate with the real particle size and might result in particle size diameter overestimation [64]. The use of more powerful SRFM techniques, such as stimulated emission depletion (STED) or stochastic optical reconstruction microscopy (STORM) could reduce this effect [65]. However, STED and STORM require specific fluorophores that tolerate the high laser intensities applied and also a higher level of expertise and equipment infrastructure in comparison to HyVolution2 SRFM. 

The PSD of EVs was assessed by NTA and flow virometry (Figure 5 and Figure 6). In both cases, EVs presented an average diameter comprised between 100 and 200 nm, similar to that of VLPs. Nonetheless, a right-skewed distribution with vesicles diameters from 45 to 500 nm was observed, especially in Sf9 samples. These results correlate with the existence of several types of EVs, encompassing exosomes and microvesicles, and BVs as visualized in cryo-TEM micrographs (Figure 3). Of note, the PSD of rod-shaped baculoviruses could not be accurately characterized by any of these methods since they assume that all nanoparticles are spherical. Van der Pol and coworkers also observed the size heterogeneity of EVs, with diameters ranging between 70 and 800 nm. They report a minimum detection limit for EVs of 70–90 nm with NTA and 150–190 nm by flow virometry [5]. Lower EV detection limits of 45 nm with NTA and 100 nm by flow virometry were reported in this work. The sample composition and also the implementation of the V-SSC instead of the 488 nm SSC in flow virometry could contribute to explain these differences [34]. Still, a higher level of accuracy is possible by using National Institute of Standards and Technology (NIST)-traceable beads in the Mie correlation with flow virometry. 

### 4.4. Particle Concentration

SRFM, NTA and flow virometry were compared for the quantification of nanoparticles in HEK 293 and Sf9 samples (Figure 4, Figure 5 and Figure 6 and Table 2). Compared to SRFM and NTA, flow virometry yielded a lower concentration of nanoparticles being around 30-fold lower for VLPs and >60-fold lower for EVs (Table 2). Similar differences in quantification by flow virometry have also been reported by van der Pol and co-authors in the analysis of urinary vesicles [5]. Differences in nanoparticle quantification between techniques could be related to the swarm effect, which is based on the fact that more than one particle passes through the detector simultaneously. However, the analysis of the same sample dilutions by flow virometry and NTA resulted in a linear correlation between both methods (VLPs, R^2^ > 0.99; total particles R^2^ > 0.91), indicating that the swarm effect could not be the main reason behind these results (Supplementary materials S3). On the other hand, it is also possible that the detection of small particles fell in the range of the background signal of the cytometer, thus contributing to reduce the final titers. Even though the average particle size of EVs and VLPs was above the minimum detection particle diameter of flow virometry, additional refinement of the Mie correlation could still be required to adequately explain these data.

Analysis of the different nanoparticle populations in each platform revealed that at least 50% of the total particles produced did not correspond to VLPs (Table 2). The possibility to discriminate but also simultaneously quantify VLPs and the rest of specimens by NTA and flow virometry represents a remarkable advantage for nanoparticle-based bioprocesses. However, further refinement of light scattered-based methodologies is required since a remarkable background signal is detected in medium solutions devoid of particles (Supplementary materials S1).

## 5. Conclusions

The selection of an adequate analytical method is essential in VLP characterization processes. Among the six methodologies studied in this work, cryo-TEM was shown as the best method to resolve nanoparticle structures while maintaining its native conformation. This technique allowed for a detailed characterization of the different EV and BV subpopulations co-produced with VLPs in HEK293 and Sf9 productions. Alternatively, the high-throughput analysis of VLPs and their differentiation from other contaminant particles was achieved by flow virometry, SRFM and NTA. Among them, flow virometry showed to be the fastest method for PSD analysis while also allowing to simultaneously quantify different nanoparticle subpopulations. Nonetheless, further improvements of these methods are required since different quantification results are observed between techniques.

## Figures and Tables

**Table 1:** NTA analysis settings.

	VLPs		Total Nanoparticles	
	Camera Level	Threshold	Camera Level	Threshold
HEK 293 supernatants	16	4	10	4
Sf9 supernatants	16	3	8	3
HEK 293 conditioned medium	-	-	10	4
Sf9 conditioned medium	-	-	14	3
FreeStyle culture medium	-	-	13	5
Sf900III culture medium	-	-	13	4

**Table 2:** Quantification of VLP and EV concentrations in transfected HEK 293 and BV infected Sf9 supernatants using different analytical methods.

Technique	VLPs (10^9^/mL)			EVs (10^9^/mL)	
	FV	NTA	SRFM	FV	NTA
HEK 293	0.2 ± 0.0	5.0 ± 0.5 *	4.0–43.0 *	0.2 ± 0.0	16.6 ± 2.9 *
Sf9	0.2 ± 0.0	8.6 ± 2.1 *	6.6–74.5 *	0.3 ± 0.0	9.7 ± 1.6 *
Fold difference (Sf9:HEK 293)	1.5	1.7	1.7	1.2	0.6

* From González-Domínguez et al. [24].

**Table 3:** Comparison of the different analytical technologies assessed in this work.

Parameter	TEM	SEM	Cryo-TEM	HyVolution2 SRFM	NTA	Flow Virometry
Expertise	+++	+++	+++++	+++	++	++
Time of measurement	++++	++++	+++++	+++	++	+
Resolution	Nanometric (<10 nm) [14]	Nanometric (<10 nm) [48]	Atomic [13]	Nanometric (140 nm) [26]	Nanometric (30 nm) [29]	Nanometric (100–200 nm) [5]
Pros	Widely used, ultrastructural analysis	Surface analysis of nanoparticle populations	Ultrastructural analysis in its native form	Direct visualization, compatible with fluorescence	Easy handling, compatible with fluorescence	Simultaneous analysis of light scattering and fluorescence labelling
Cons	Staining required, working under vacuum conditions	Working under vacuum conditions	Time consuming, working at cryogenic temperatures	Data analysis, convolution effect of light	Screening effect, variability due to acquisition settings	Sensitivity, inter-equipment variability

## Supplementary Materials

The following are available online at https://www.mdpi.com/1999-4915/12/2/223/s1, Figure S1: Negative control analyses: cell culture and conditioned media controls, Figure S2: Mie correlation for EV analysis with FCM_PASS_ software, Figure S3: Nanoparticle correlation between flow virometry and NTA.

## Abbreviations

AB	Alcian Blue
AFM	Atomic Force Microscopy
BV	Baculovirus
CQA	Critical Quality Attributes
Cryo-TEM	Cryogenic Transmission Electron Microscopy
DLS	Dynamic Light Scattering
EM	Electron Microscopy
EV	Extracellular Vesicle
cBV	filled baculovirus with vesicle-like or protein content
LM	Lipid Membrane
MVB	Multivesicular Body
NS	Negative Staining
NTA	Nanoparticle Tracking Analysis
ODV	Occlusion-Derived Baculovirus
PSD	Particle Size Distribution
RI	Refractive Index
rBV	relaxed-form of baculovirus
RT	Room Temperature
SEM	Scanning Electron Microscopy
SRFM	Super-Resolution Fluorescence Microscopy
TEM	Transmission Electron Microscopy
TRPS	Tunable Resistive Pulse Sensing
V-SSC	Violet Side Scatter
VLP	Virus-Like Particle
