# Supplementary material for: Quality Assessment of Virus-Like Particles at Single Particle Level: A Comparative Study
Source: Viruses. 2020 Feb 17;12(2):223. doi: 10.3390/v12020223 (PMC7077327; doi:10.3390/v12020223)
Supplement: Supplementary file 1 [file viruses-12-00223-s001.pdf]

SUPPLEMENTARY MATERIALS

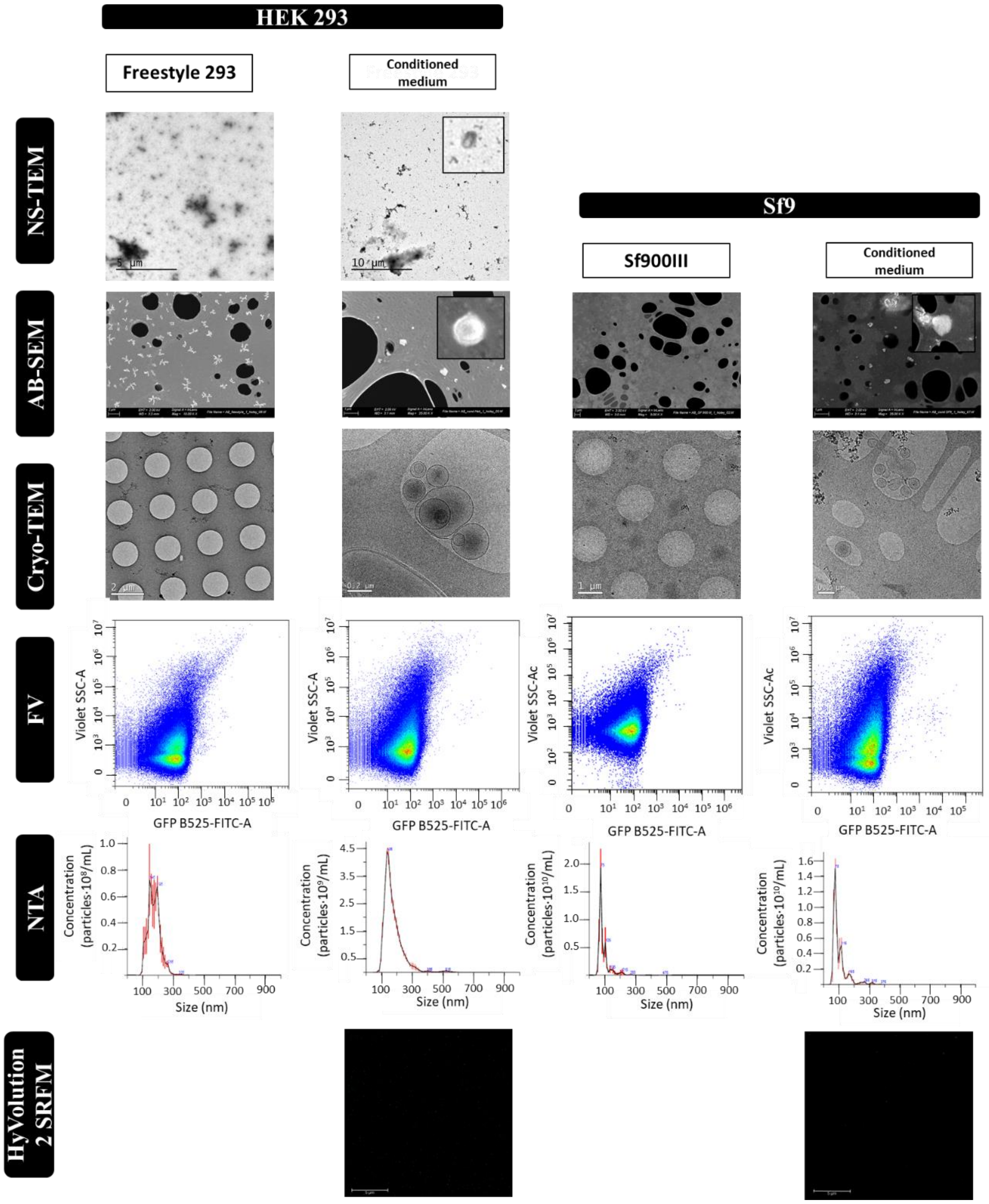

S1. Negative control analyses: cell culture and conditioned media controls. FreeStyle 293 (HEK 293) and Sf900III (Sf9) media were assessed by NS-TEM, AB-SEM, cryo-TEM, NTA, flow virometry and SRFM. Analyses were performed by applying the same sample preparation protocol and equipment set up that used for VLP samples. In NS-TEM, AB-SEM and cryo-TEM analyses of FreeStyle medium, the presence of salts was detected while low electrodense aggregates were observed in the Sf900III medium by cryo-TEM. In EM micrographs of both conditioned media, the presence of EVs was detected. NTA analyses of FreeStyle and Sf900III media, and conditioned media resulted in a mean nanoparticle diameter of 174 ± 39 nm (FreeStyle), 174 ± 65 nm (conditioned FreeStyle), 92.7 ± 74.6 nm (Sf900III), 101 ± 47 nm (conditioned Sf900III) with a respective concentration of 0.6 ± 0.1 E+9, 17.6 ± 0.9 E+9, 24.4 ± 1.1

E+9 and 27.3 ± 1.5E+9 diffracting particles/ml. Conditioned media analyses are adapted from [24] and FreeStyle medium results from [31]. Flow virometry analyses of FreeStyle medium, conditioned FreeStyle, Sf900III medium and conditioned Sf900III yielded a nanoparticle concentration of 0.1 E+9, 0.2 E+9, 0.1 E+9, 0.8 E+9 of EVs, respectively. No particles were detected by HyVolution2 SRFM analysis of FreeStyle and Sf900III conditioned media samples due to the absence of HIV-1 Gag-eGFP VLPs, as expected. This data was adapted from [24].

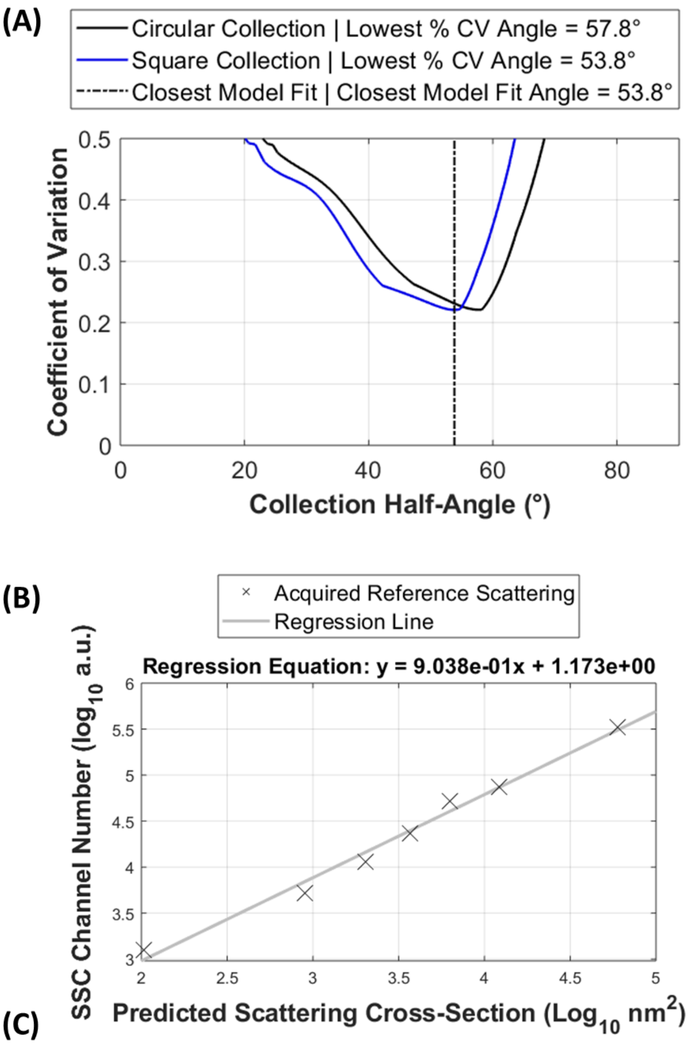

(C)

Bead Diameter (nm)	Median V-SCC (a.u.)	rCV (%)
100	1246.4	18.9
160	5248.8	10.3
200	11542.3	3.5
240	23266.4	6.5
300	51993.8	2.6
500	75025.8	3.8
900	328552.7	2.4

S2. Mie correlation for EV analysis with FCMPASS software. (A) Collection half-angle plot. (B) Lineal regression SSC vs. predicted scattering cross-section plot. (C) Megamix beads information acquired with the CytoFlex LX and introduced in the FCMPASS software.

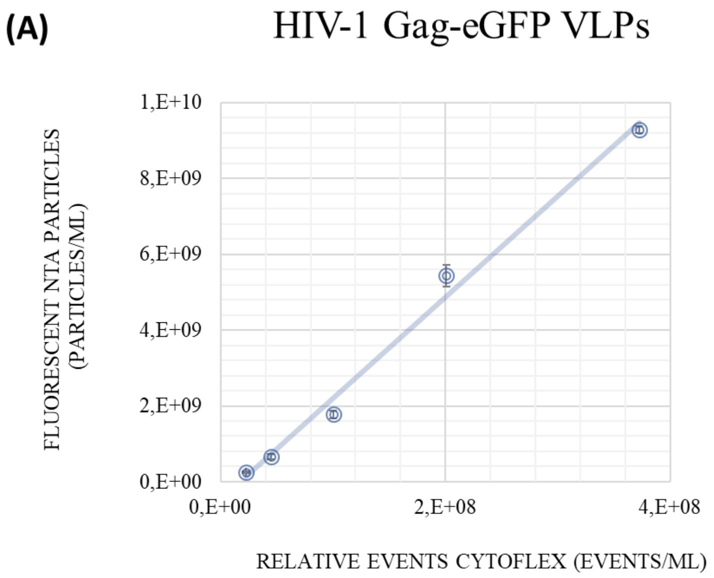

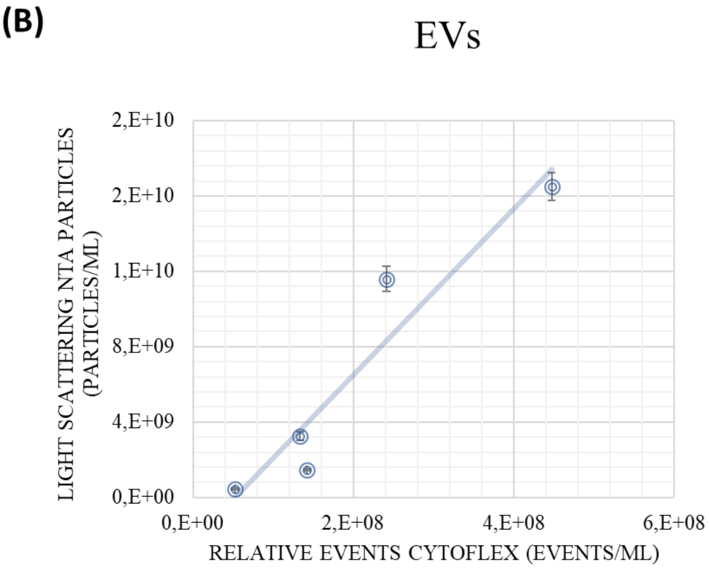

S3. Correlation between flow virometry and NTA of nanoparticles produced in HEK 293 cells. (A) HIV-1 Gag-eGFP VLPs (fluorescent particles) measured with NTA using the fluorescence filter and the population corresponding to VLPs in flow virometry are represented. Regression: (Fluorescent particles/mL) = 26,67 (events /mL) - 5E+08; R^2 = 0,9909. (B) EVs measured with light scattering by NTA and the population corresponding to EVs in flow virometry are represented. Regression: (Light Scattering particles/mL) = 44,18 (events /mL) - 2E+09; R^2 = 0,9082.
